# Down-regulation of microRNA-203a suppresses IL-1β-induced inflammation and cartilage degradation in human chondrocytes through Smad3 signaling

**DOI:** 10.1042/BSR20192723

**Published:** 2020-03-13

**Authors:** Yongbo An, Guang Wan, Jingang Tao, Mingxing Cui, Qinglan Zhou, Wengen Hou

**Affiliations:** 1 Department of Orthopedics II, The First Affiliated Hospital of Xinxiang Medical University, Weihui 453100, Henan, China; 2 Department of Orthopedics IV, The First Affiliated Hospital of Xinxiang Medical University, Weihui 453100, Henan, China; 3 Department of Orthopedics I, The First Affiliated Hospital of Xinxiang Medical University, Weihui 453100, Henan, China

**Keywords:** articular cartilage, ECM, inflammatory cytokines, miR-203a, Osteoarthritis

## Abstract

Osteoarthritis (OA) is a chronic and prevalent degenerative musculoskeletal disorder, which is characterized by articular cartilage degradation and joint inflammation. MicroRNA-203a (miR-203a) has been shown to be involved in multiple pathological processes during OA, but little is known about its function in chondrocyte extracellular matrix (ECM) degradation. In the present study, we aimed to elucidate the effects of miR-203a on articular cartilage degradation and joint inflammation. We observed that the miR-203a level was significantly up-regulated in OA tissues and in an *in vitro* model of OA, respectively. Inhibition of miR-203a significantly alleviated the interleukin (IL)-1β-induced inflammatory response and ECM degradation in chondrocytes. Moreover, mothers against decapentaplegic homolog 3 (Smad3), a key factor in maintaining chondrocyte homeostasis, was identified as a putative target of miR-203a in chondrocytes. More importantly, inhibition of Smad3 impaired the inhibitory effects of the miR-203a on IL-1β-induced inflammatory response and ECM degradation. Collectively, these results demonstrated that miR-203a may contribute to articular cartilage degradation of OA by targeting Smad3, suggesting a novel therapeutic target for the treatment of OA.

## Introduction

Osteoarthritis (OA) is the most common chronic joint disease, which is characterized by articular cartilage degradation, joint inflammation and secondary bone hyperplasia [1]. Chondrocytes are the only cell type present in articular cartilage and play a key role in tissue homeostasis and maintaining matrix integrity [2]. As the progress of OA, the chondrocytes are metabolically activated and increase apoptosis and pro-inflammatory cytokine secretion, and lead to continuous loss of extracellular matrix (ECM) [3]. Therefore, targeting the chondrocyte injury is effective prevention and therapeutic methods in the development of OA.

MicroRNAs (miRNAs) are endogenous small noncoding RNAs of 18–24 nucleotides in length, which act as a unique regulator of gene expression at the post-transcriptional level by inhibiting translation or promoting RNA degradation [4]. Expectedly, more and more miRNAs are found to be abnormally expressed in human OA cartilage tissue, among which some have been shown to play a role in the pathological processes of OA [5,6]. Several miRNAs have also been reported to regulate inflammation and articular cartilage degradation in OA, such as miR-138 and miR-101. It was also shown that microRNA-203a (miR-203a) modulated inflammatory reaction in different inflammatory diseases [7]. Recently, Luan and Liang [8] demonstrated that inhibition of miR-203a alleviated lipopolysaccharide-induced inflammatory injury in murine chondrogenic ATDC5 cells. However, miR-203a regulates ECM in the development of OA is not reported.

In the present study, we investigated the miRNA expression profiles in human OA articular cartilage tissues. Subsequently, the role of miR-203a in interleukin (IL)-1β-treated chondrocytes and the potential effects of this miRNA on the cellular processes of articular cartilage degradation and inflammation were assessed. Our findings provide valuable insight into the complex coordinated regulation of IL-1β-induced apoptosis, cartilage degradation and inflammation by miR-203a in chondrocytes, which may lead to novel therapeutic strategies for OA.

## Materials and methods

### Clinical samples

Human cartilage tissues were collected from OA patients (*n*=30) who underwent total knee replacement surgery and from traumatic amputees (*n*=20) without rheumatoid arthritis or OA. OA patients were diagnosed according to the American College of Rheumatology criteria [9,10]. All tissues were evaluated using Safranin O-fast green staining and graded according to a modified Mankin scale (normal, scored 0–2; moderate OA, 6–10; and severe OA, 11–14) [11]. A total of 30 OA patients, 11 moderate and 19 severe OA patients were identified. All samples were obtained from the Department of Orthopedics II, The First Affiliated Hospital of Xinxiang Medical University. Written informed consent was obtained from all patients and the study was approved by the Ethics Committee of Xinxiang Medical University.

### Cell culture

Human chondrocytes were isolated from the OA knee joints as previously described [12]. Chondrocytes were grown in Dulbecco’s modified Eagle’s medium (DMEM, Life Technologies) containing 10% (v/v) fetal bovine serum (FBS, Gibco BRL, Grand Island, NY) and 1% penicillin/streptomycin (Sigma, St. Louis, MO). First passage chondrocytes were released from cartilage and cultured in high density for 1 week. All experiments were done within 3 days after passage 1 culture, and dedifferentiation was minimized by the duration of monolayer culture. Cultures were maintained in a humidified incubator at 37°C with 5% CO_2_ or 95% air, and the medium was changed every 2–3 days.

### Cell transfection

The specific miR-203a mimics/inhibitor and corresponding negative control (NC), as well as specific small interfering RNA for Smad3 (si-Smad3) and si-Scramble were designed and purchased from GenePharma Co., Ltd (Shanghai, China). The cells were transfected with these oligo fragments using Lipofectamine 2000 (Invitrogen, Carlsbad, CA, U.S.A.) according to the manufacturer’s instructions. At 48 h after transfection, cells were stimulated with IL-1β (5 ng/ml) for the indicated time and used for further analysis.

### miRNA microarray analysis

miRNA microarray analysis was performed to identify miRNA expression profiles in human cartilage tissues. Total RNA was isolated using TRIzol reagent (Molecular Research Center, Inc., Cincinnati, OH, U.S.A.), and mirVana miRNA isolation kit (Ambion, Austin, TX) was used to further purify miRNA fraction according to the manufacturer’s instructions. The isolated miRNAs were labeled with Hy3 using the miRCURY array labeling kit (Exiqon, Vedbaek, Denmark) and hybridized with miRCURY locked nucleic acid (LNA) microRNA arrays (v8.0; Exiqon). Replicated miRNAs were averaged, and miRNAs with intensities ≥50 in all samples were used to calculate a normalization factor. Data normalization was performed using the quantiles method. Microarray images were taken with a Genepix 4000B scanner (Axon Instruments, Foster City, CA, U.S.A.) and analyzed with Genepix Pro 6.0 software (Axon Instruments).

### RNA extraction and quantitative real-time PCR

Total RNA was isolated using TRIzol Reagent (Invitrogen Corp, Grand Island, NY, U.S.A.) from cells according to manufacturer’s protocols. The Smad3 and miRNA were reverse transcribed using TaqMan Gene Expression Assays kit and TaqMan MicroRNA Reverse Transcription kit (Applied Biosystems, Thermo Fisher Scientific, CA, U.S.A.), respectively. The gene expressions of aggrecan, type II collagen and matrix metalloproteinase (MMP)-13 were measured by the SYBR Green PCR kits (TAKARA). The *U6* gene was used as a reference control for miR-203a and the GAPDH was used as a reference control for Smad3, aggrecan, type II collagen and MMP-13. Quantitative real-time PCR (qRT-PCR) was performed using an Applied Biosystems 7500 Real-Time PCR machine with miRNA-specific primers using TaqMan Gene Expression Assay (Applied Biosystems). All reactions were performed in triplicate. The miR-203a relative expression was analyzed using the 2^−ΔΔ*C*_t_^ method.

### Cell viability analysis

The cell counting kit-8 (CCK-8) assay was used to detect cell proliferation according to the manufacturer’s instructions [13]. Briefly, transfected chondrocytes were seeded into 96-well plates with complete medium at a density of 2 × 10^4^ and stimulated with IL-1β. At the indicated time point, 10 μl CCK-8 reagent (Dojindo Molecular Technologies, Inc. Rockville, MD, U.S.A.) was added for another 4 h culture at 37°C. The absorbance was measured at 450 nm using a Microplate Reader (Bio-Rad, Hercules, CA, U.S.A.).

### Apoptosis analysis

The chondrocytes were treated with IL-1β following transfection. Then, cell apoptosis was detected using annexin V-FITC (BD, Mountain View, CA, United States) and propidium iodide (PI, 50 μg/ml) (BD, Mountain View, CA, United States) staining according to the manufacturer’s instructions. Briefly, the cells were collected and washed twice with ice-cold PBS and then resuspended in binding buffer. The cells were then cultured with 5 μl Annexin V-FITC and 10 μl PI at room temperature in the dark for 20 min. Stained cells were analyzed using flow cytometry (BD, FACSCalibur, CA, United States). The measurements were taken independently at least three times with similar results.

### Enzyme-linked immunosorbent assay

Inflammatory cytokines, containing tumor necrosis factor α (TNF-α), IL-1β and IL-6 in supernatant samples were measured using Enzyme-linked immunosorbent assay (ELISA) kits (R&D Systems, Minneapolis, MN, U.S.A.) according to the manufacturer’s instructions.

### Immunofluorescence assay

Cells were rinsed in PBS three times and fixed with 4% formaldehyde in PBS for 15 min at room temperature. After that, goat serum was used to block nonspecific binding sites. The cultured cells were then incubated with an MMP-13 antibody (Abcam; 1:200 dilution) in PBS for 8 h at 4°C. After three washes with PBS, each for 3 min at room temperature, the cells were incubated for 1 h with a Cy3-conjugated goat anti-rabbit IgG antibody (1:100 dilution, Sigma–Aldrich, St. Louis, MO, U.S.A.) for 1 h, and counterstained with DAPI. After the final round of three washes, the samples were mounted on slides and examined under a confocal microscope (Nikon Eclipse 80i; Nikon, Tokyo, Japan).

### Luciferase assay

The miR-203a mimics/inhibitor and corresponding NC were purchased from GenePharma (Shanghai, China). The potential binding site between Smad3 and miR-203a was searched using TargetScan (Figure 4A) (http://www.targetscan.org). The Smad3 3ʹ-UTR with wild-type (wt) and mutant (mut) containing the putative binding site of miR-203a were constructed (Figure 4B) and subsequently cloned into a pMIR-REPORT luciferase reporter vector (Ambion, U.S.A.). The mutated plasmid, pMIR-Smad3-mut-3ʹ-UTR was generated by a QuikChange Kit (Qiagen). The chondrocytes were co-transfected with 400 ng of the reporter construct (pMIR-Smad3-3ʹ-UTR or pMIR-Smad3-3ʹ-UTR) and 50 ng miR-203a mimic/inhibitor or corresponding NC using Lipofectamine 2000 reagent (Invitrogen). Forty-eight hours after transfection, the relative firefly luciferase activity normalized with *Renilla* luciferase was measured using the Dual-Light luminescent reporter gene assay (Applied Biosystems).

### Western blot analysis

The cells were lysed as described previously [14]. The protein concentration was quantified using BCA protein assay kit (Pierce, Rockford, IL). The protein samples (60 μg) were separated in 10% SDS/PAGE gel (Sigma–Aldrich, St. Louis, MO) and then transferred to polyvinylidene difluoride (PVDF) membranes (Bio-Rad, Richmond, CA, U.S.A.). The membrane was blocked by 5% skimmed milk at room temperature for 1 h, and incubated primary antibodies against Smad3 (Santa Cruz Biotechnology, Santa Cruz, CA, U.S.A.) overnight at 4°C. β-actin (Sigma, St. Louis, MO, U.S.A.) served as an internal control. Horseradish peroxidase-conjugated (HRP) antibodies (Santa Cruz Biotechnology, Santa Cruz, CA, U.S.A.) were used as the secondary antibodies. Subsequently, the protein bands were scanned on the X-ray film using the enhanced chemiluminescence detection system (PerkinElmer Life and Analytical Sciences, Boston, MA). The band intensity was quantified by alpha Imager software (Alpha Innotech Corporation, San Leandro, CA). The measurements were carried out independently for at least three times with similar results.

### Statistical analysis

All statistical analyses were performed using SPSS 14.0 software (Chicago, IL). Each experiment was repeated at least three times. Numerical data are presented as the means ± SD. A one-way analysis of variance (ANOVA) followed by post-hoc tests were used to verify statistically differences among the groups. *P*-value of <0.05 was considered significant and <0.01 was considered very significant.

## Results

### miR-203a is overexpressed in human cartilage tissues and IL-1β-induced chondrocytes

**Figure 1:**
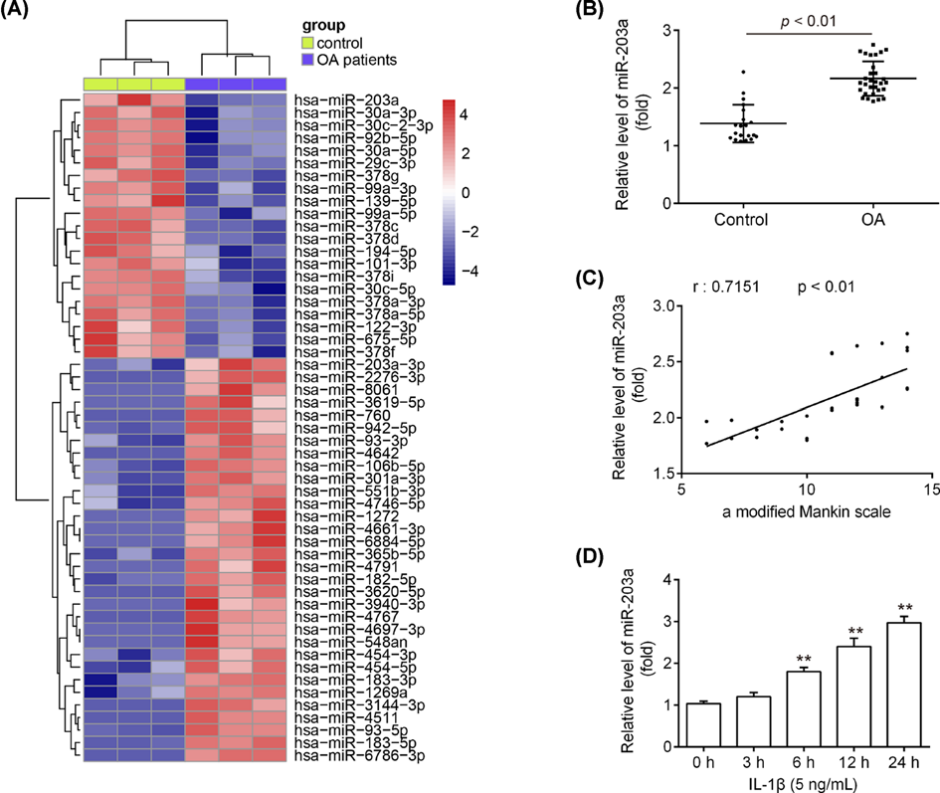
The miR-203a level in human OA articular cartilage tissues and IL-1β-induced chondrocytes (**A**) The microarray analysis was used to identify the miRNA expression profiles in human normal and OA articular cartilage tissues. Red or green color separately shows high or low expression in the heatmap. (**B**) The miR-203a level was determined by qRT-PCR in human normal (*n*=20) and OA articular cartilage tissues (*n*=30). (**C**) The miR-203a expression level was positively correlated with Mankin scale (r = 0.7151, *P*<0.01). (**D**) Chondrocytes were treated with IL-1β at 5 ng/ml for 0, 3, 6, 12, or 24 h, and the miR-203a level were detected using qRT-PCR. Data are presented as means ± SD of three individual experiments (**P<0.01 vs. control).

To investigate the possible role of miRNAs in the OA processes, we performed the miRNA microarray analysis to detect the miRNA expression profiles in human normal and OA articular cartilage tissues. As shown in Figure 1A, a large set of miRNAs were abnormally expressed in OA articular cartilage tissues, and miR-203a was one of the most dramatically up-regulated miRNAs. Moreover, qRT-PCR further confirmed that miR-203a level was obviously higher in OA articular cartilage tissues than normal tissues (Figure 1B; *P*<0.01). Importantly, the miR-203a expression was positively correlated with a modified Mankin scale (Figure 1C; r = 0.7151, *P*<0.01). Subsequently, IL-1β (5 ng/ml) was used to simulate inflammatory injury in chondrocytes and the miR-203a levels were determined by qRT-PCR. Compared with untreated controls, our results exhibited that IL-1β could significantly increase the miR-203a expression levels in chondrocytes, and this promoting effect was time-dependent (Figure 1D; *P*<0.01). These results indicated that miR-203a may be involved in the pathology of OA.

### Knockdown of miR-203a alleviates IL-1β-induced cell viability reduction, cell apoptosis and inflammatory cytokines production

**Figure 2:**
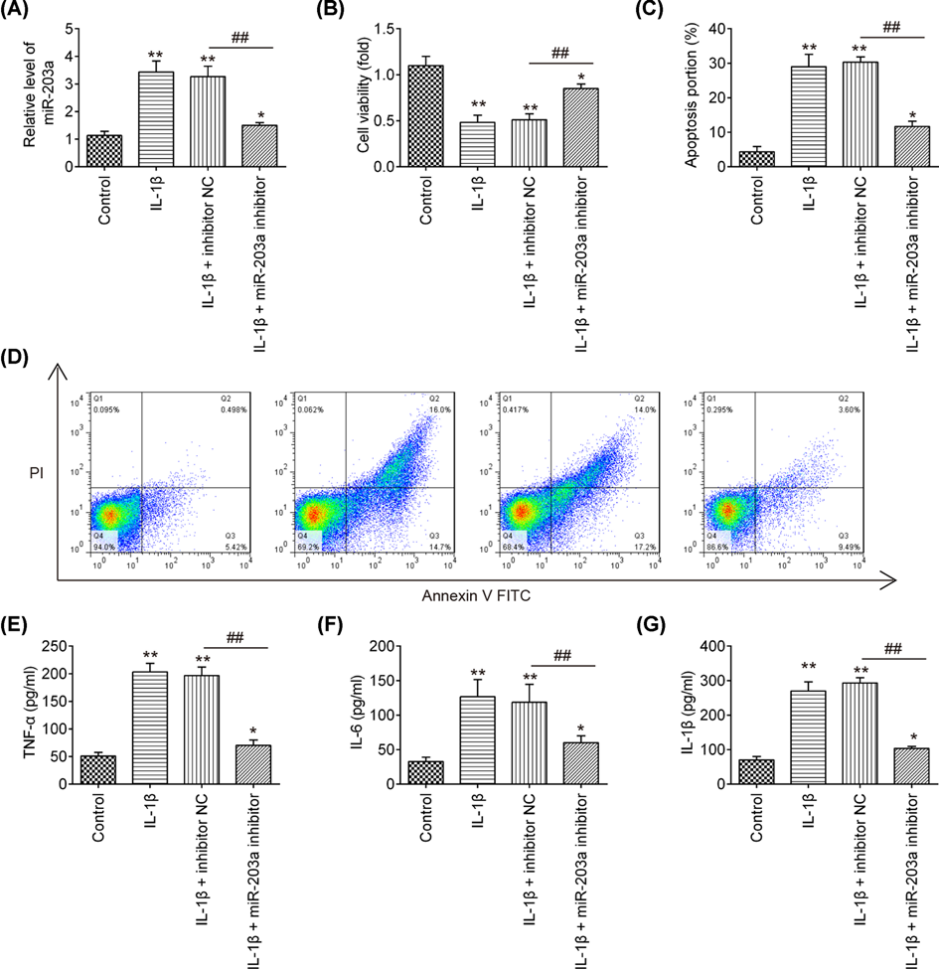
The effect of miR-203a down-regulation on the IL-1β-induced cell viability, apoptosis and inflammatory cytokines production Chondrocytes transfected with miR-203a inhibitor and inhibitor NC and then treated with IL-1β at 5 ng/ml for 24 h. (**A**) The miR-203a mRNA level was measured by the RT-qPCR assay. (**B**) The CCK-8 assay was used to measure the cell viability. (**C,D**) Cell apoptosis was evaluated using flow cytometry. (**E**–**G**) Inflammatory cytokines TNF-α, IL-1β and IL-6 in supernatant samples were detected using ELISA kits, respectively. Data are presented as means ± SD of three individual experiments (*P<0.05, **P<0.01 vs. control group, ^##^*P*<0.01 vs. IL-1β + inhibitor NC).

To explore the role of miR-203a in IL-1β treated chondrocytes, we transfected miR-203a inhibitor into the OA chondrocytes and then stimulated the cells with IL-1β. As shown in Figure 2A, miR-203a was notably decreased in chondrocytes after miR-203a inhibitor transfection, which confirmed the transfection efficiency (*P*<0.01). Subsequently, the cell viability, apoptosis and inflammatory cytokines production were examined. The results demonstrated that IL-1β stimulation significantly inhibited cell viability and increased apoptosis, whereas knockdown of miR-203a reversed these effects of IL-1β in chondrocytes (Figure 2B–D; *P*<0.01). Furthermore, the increased inflammatory cytokines, including TNF-α, IL-6 and IL-1β induced by IL-1β were all dramatically reduced by down-regulation of miR-203a (Figure 2E–G; *P*<0.01). These data suggested that inhibition of miR-203a attenuates IL-1β-induced cell apoptosis and inflammatory response in chondrocytes.

### Knockdown of miR-203a alleviates the IL-1β-induced ECM metabolic imbalance

**Figure 3:**
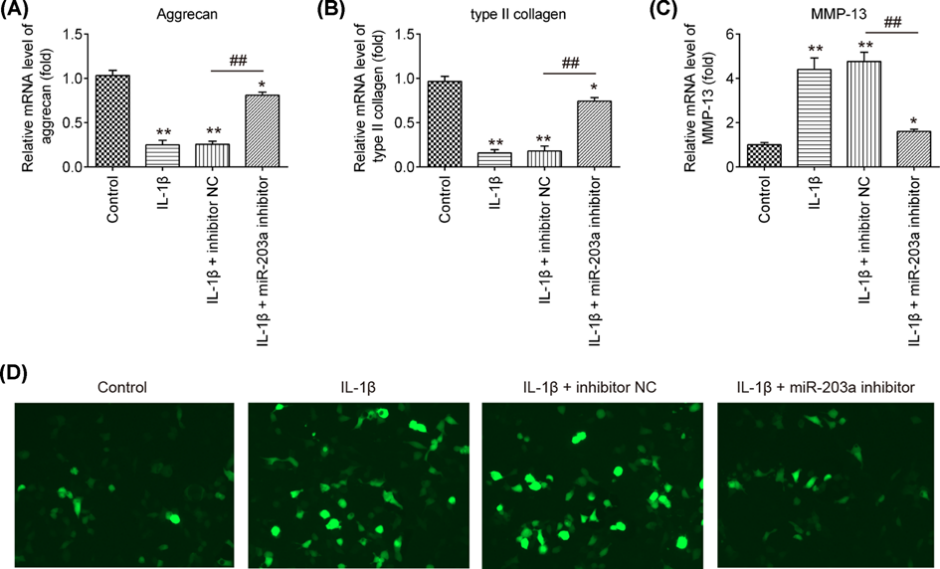
The effect of miR-203a down-regulation on the IL-1β-induced aggrecan, type II collagen and MMP-13 The chondrocytes were transfected with miR-203a inhibitor or inhibitor NC, and then exposed chondrocytes to IL-1β for 24 h. (**A**–**C**) The mRNA levels of aggrecan, type II collagen and MMP-13 were analyzed using qRT-PCR. (**D**) The MMP-13 protein level was detected using immunofluorescence assay. Data are presented as means ± SD of three individual experiments (*P<0.05, **P<0.01 vs. control group, ^##^*P*<0.01 vs. IL-1β + inhibitor NC).

ECM metabolic factors contribute to the pathogenesis of OA, which is predominantly composed of Col II, aggrecan and MMPs are primary enzymes responsible for ECM degradation [15]. To further investigate whether miR-203a effects IL-1β-induced ECM degradation of OA chondrocytes, we used qRT-PCR to examine the expression change of aggrecan, type II collagen and MMP-13. We observed that IL-1β stimulation dramatically led to a reduction in aggrecan and type II collagen and an increase in MMP-13 compared with control group, nevertheless knockdown of miR-203a significantly reversed the IL-1β-induced effects on aggrecan, type II collagen and MMP-13 (Figure 3A–C; *P*<0.01). We also found the up-regulated MMP-13 protein level induced by IL-1β was obviously inhibited in the cells transfected with miR-203a inhibitor, as determined by IFA (Figure 3D). These results indicated that down-regulation of miR-203a ameliorates the IL-1β-induced ECM degradation in chondrocytes.

### Smad3 is a direct target of miR-203a in chondrocytes

**Figure 4:**
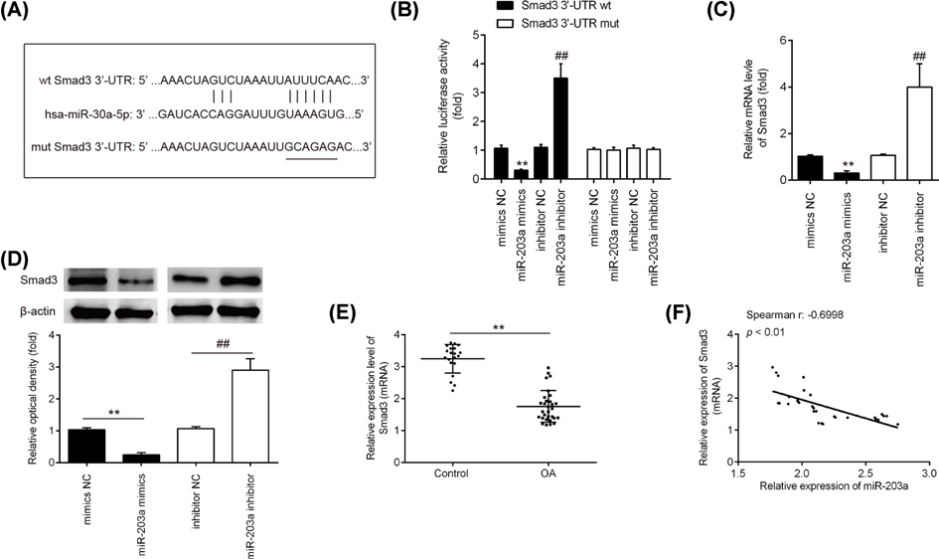
Smad3 is a direct target of miR-203a in chondrocytes (**A**) The Smad3 3ʹ-UTR region containing the wild-type (wt) or mutant (mut) binding site for miR-203a. (**B**) The chondrocytes were co-transfected with the reporter construct (pMIR-Smad3-3ʹ-UTR or pMIR-Smad3-3ʹ-UTR) and miR-203a mimic/inhibitor or corresponding NC and the relative luciferase activity were measured (**P<0.01 vs mimic NC; ^##^*P*<0.01 vs. inhibitor NC). (**C**) The Smad3 mRNA level was detected after miR-203a mimics transfection by qRT-PCR. (**D**) The protein level of Smad3 was detected using Western blot analysis (**P<0.01 vs. mimic NC; ^##^*P*<0.01 vs inhibitor NC). β-actin was used as an internal control. (**E**) The Smad3 mRNA level was detected using qRT-PCR in the OA articular cartilage tissues (*n*=30) and normal tissues (*n*=20) (**P<0.01 vs. control). (**F**) The negative correlation between Smad3 and miR-203a levels in the OA articular cartilage tissues (r = −0.6998, *P*<0.01). Data are presented as means ± SD of three individual experiments.

To investigate the potential molecular mechanism of miR-103a-regulated inflammatory events and ECM degradation in OA, we performed TargetScan (http://www.targetscan.org) to predicate the putative targets of miR-203a. A putative miR-203a binding site was detected within the 3ʹ-UTR of the Smad3 mRNAs, as indicated in Figure 4A. To determine whether a direct interaction occurs between miR-203a and its putative binding sites in the 3ʹ-UTRs of Smad3 mRNA, a luciferase reporter assay was performed in chondrocytes. The luciferase reporter gene assay showed that miR-203a overexpression markedly repressed the luciferase activity, whereas the miR-203a knockdown increased the relative luciferase activity of constructs containing the wt Smad3 3ʹ-UTR (*P*<0.01; Figure 4B). Expectedly, miR-203a mimics/inhibitor failed to regulate the relative luciferase activity of vector containing mut Smad3-3ʹ-UTR in the miR-203a-binding site (Figure 4B). Subsequently, the effect of miR-203a on the expression of Smad3 was measured at the mRNA and protein level in chondrocytes by qRT-PCR and Western blot analysis. It was shown that overexpression of miR-203a significantly inhibited the mRNA and protein expression level of Smad3 (*P*<0.01; Figure 4C,D), while knockdown of miR-203a enhanced the Smad3 expression levels in chondrocytes (*P*<0.01; Figure 4C,D). As miR-203a was up-regulated in OA articular cartilage tissues, the expression levels of Smad3 were also detected in these tissues by qRT-PCR. The results showed that Smad3 expression was significantly decreased in the OA articular cartilage tissues compared with normal tissues (*P*<0.01; Figure 4E). Correlation analysis also showed an inverse correlation between miR-203a level and Smad3 expression in the OA articular cartilage tissues (r = −0.6998, *P*<0.01; Figure 4F). Taken together, these data suggested that miR-203a has a modulatory effect of Smad3 expression.

### Smad3 silencing rescues the effects of miR-203a down-regulation on IL-1β-stimulated chondrocytes

**Figure 5:**
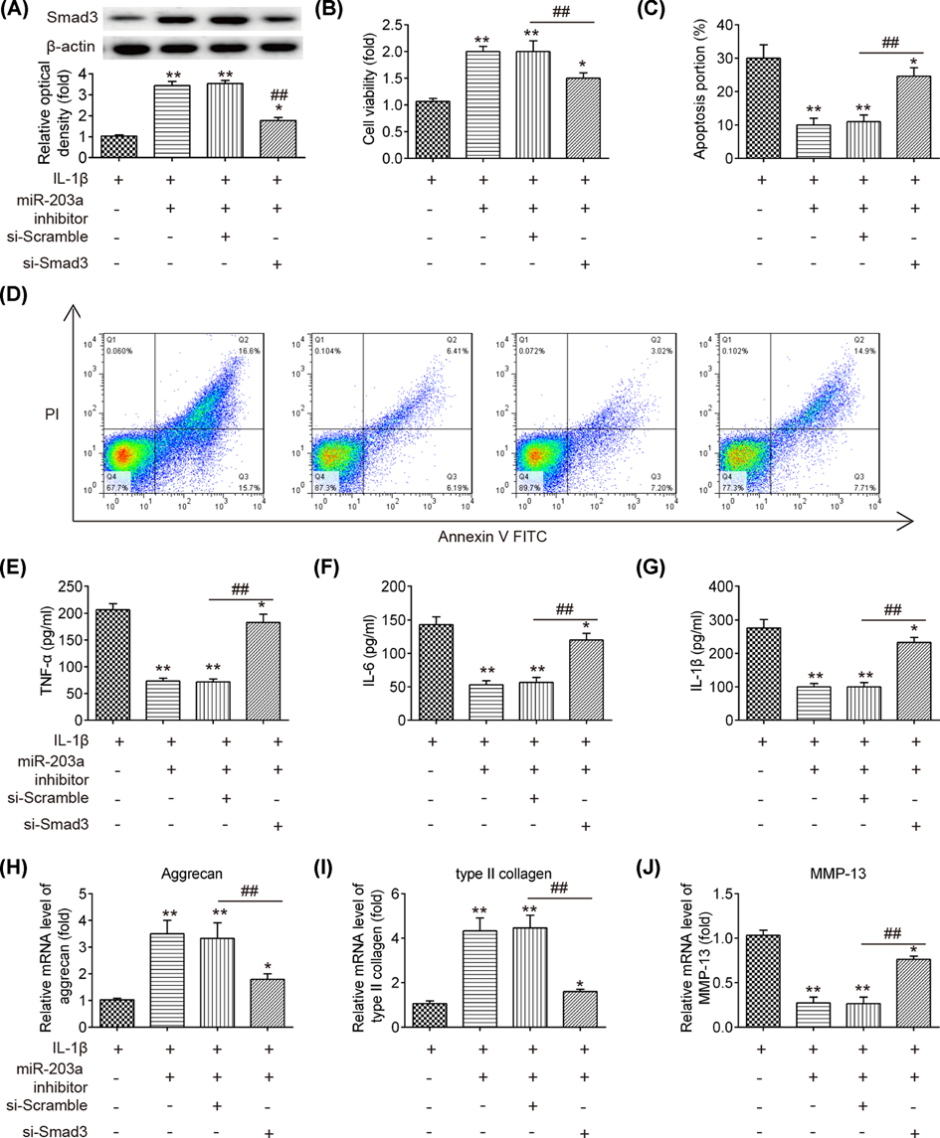
Smad3 silencing rescues the effects of miR-203a down-regulation on IL-1β-stimulated chondrocytes The chondrocytes were transfected with miR-203a inhibitor or were co-transfected with miR-203a inhibitor and si-Smad3 or si-Scramble, and then treated with IL-1β at 5 ng/ml for 24 h. (**A**) The Smad3 protein level was determined using Western blot analysis. (**B**) Cell viability was measured using CCK-8 assay. (**C,D**) The flow cytometry was conducted to measure cell apoptosis. (**E**–**G**) Inflammatory cytokines TNF-α, IL-1β and IL-6 were detected using ELISA kits, respectively. (**H**–**J**) The mRNA levels of aggrecan, type II collagen and MMP-13 were analyzed using qRT-PCR. Data are presented as means ± SD of three individual experiments (*P<0.05, **P<0.01 vs. IL-1β group, ^##^*P*<0.01 vs. miR-203a inhibitor + si-Scramble).

To investigate whether Smad3 mediated the inhibitory effects of miR-203a on inflammatory response and ECM degradation, the chondrocytes were co-transfected with miR-203a inhibitor and si-Smad3, and then treated with IL-1β at 5 ng/ml for 24 h. The transfection efficiency of si-Smad3 was determined using Western blot analysis. Smad3 protein expression levels were notably decreased in IL-1β-stimulated chondrocytes (*P*<0.01; Figure 5A). Subsequently, we found that down-regulation of miR-203a alleviated IL-1β-induced cell viability reduction, cell apoptosis and inflammatory cytokines production, whereas Smad3 silencing could reverse these inhibitory effects of miR-203a (*P*<0.01; Figure 5B–G). Moreover, knockdown of miR-203a attenuated the IL-1β-induced decrease in aggrecan and type II collagen mRNA expressions and the increase in MMP-13 mRNA expression, but these effects were blocked by si-Smad3 (*P*<0.01; Figure 5H–J). These results indicated that knockdown of miR-203a suppressed IL-1β induced inflammatory response and ECM degradation in chondrocytes via up-regulating Smad3.

## Discussion

OA, a disorder of cartilage and per-articular bone, is one of the most common causes of pain and disability worldwide [16,17]. Recent study demonstrated that more than 25 miRNAs are involved in OA and many are functionally implicated in the pathogenesis of the disease [18]. In the present study, we revealed that miR-203a was up-regulated in OA articular cartilage tissues and IL-1β-induced chondrocytes. More importantly, we found that miR-142-5p targets Smad3 and plays a crucial role in OA progression, which includes promoting cell proliferation, decreasing cell apoptosis and reducing inflammatory factors release. These findings indicate that miR-203a may be a potential target for therapeutic strategies of OA.

Recent research revealed that many miRNAs were identified to be aberrantly expressed in normal and OA chondrocytes, and play a vital role in cartilage function [19,20]. Several miRNAs have been demonstrated to be associated with the processes of disrupted cartilage homeostasis in response to IL-1β [21–23]. For example, Xiang et al. [24] have shown that miR-142-5p overexpression can inhibit apoptosis, inflammation and matrix catabolism through inactivating the MAPK signaling pathway in OA chondrocytes. Baek et al. [25] have revealed that miR-449a knockdown promoted cartilage regeneration and prevented progression of OA in *in vivo* rat models. Previous studies showed that miR-203a played important roles in cell proliferation and tumor development [26–28]. In this study, we demonstrated a novel role of miR-203a during ECM degradation and inflammation in OA. We found that miR-203a was significantly up-regulated in OA articular cartilage tissues compared with normal tissues. More importantly, inhibition of miR-203a ameliorated IL-1β-induced cell viability reduction, cell apoptosis and inflammatory cytokines production. These results indicated that miR-203a might alleviate OA pathology through suppressing chondrocyte apoptosis and inflammation.

Articular cartilage ECM is important for the repair and homeostasis of cartilage, which is predominantly composed of Col II and aggrecan [29]. Progressive loss of Col II and aggrecan is thought to be a main pathological feature of OA [30]. In addition, the synthesis of the MMPs in the chondrocytes, including MMP-1, MMP-3 and MMP-13 are primary enzymes responsible for ECM degradation [31,32]. Given the broad biological functions of miRNAs, it is not surprising that miR-203a is involved in regulation of Col II, aggrecan and MMP expression within the articular cartilage. In the present study, our results showed that IL-1β inhibited aggrecan and type II collagen expression levels, which were efficiently alleviated by the knockdown of miR-203a. Moreover, IL-1β dramatically enhances the expression of MMP-13 mRNA and protein, whereas these effects are reversed by inhibition of miR-203a. These results suggested that knockdown of miR-203a suppressed ECM degradation induced by IL-1β in chondrocytes.

Transforming growth factor-β (TGF-β), a pleiotropic cytokine/growth factor, which has anabolic effects on chondrocytes [33]. Through putative target prediction, the present study identified that miR-203a may target Smad3, which is an important component of the TGF-β signaling pathway. Previous studies have reported that mutation of Smad3 leads to cartilage degeneration and OA [34,35]. Smad3 modulates the balance between the synthesis and degradation of ECM of articular chondrocyte through decreasing MMP-13 level and increasing aggrecan and type II collagen expression [36]. In this study, we identified that miR-203a inhibits Smad3 expression by directly binds to its 3ʹ-UTR in chondrocytes. Correlation analysis also showed an obviously negative correlation between miR-203a level and Smad3 expression in the OA articular cartilage tissues. Moreover, we found that inhibition of miR-203a alleviated IL-1β-induced cell viability reduction, cell apoptosis, inflammatory cytokines production (TNF-α, IL-6 and IL-1β) and ECM metabolic imbalance, whereas these effects were blocked by Smad3 knockdown. These results demonstrated that knockdown of miR-203a exerted the protective effect on IL-1β-induced chondrocyte injury through targeting Smad3.

In summary, our results demonstrated that miR-203a is overexpressed in human cartilage tissues and IL-1β-induced chondrocytes. Down-regulation of miR-203a alleviated OA pathology by suppressing inflammatory cytokines and ECM metabolic imbalance possibly through direct targeting of Smad3. Our work provides important insight into targeting miR-203a/Smad3 axis for developing potential therapeutic strategies for OA patients.

## Abbreviations

CCK-8: cell counting kit-8
ECM: extracellular matrix
IL: interleukin
miRNA: microRNA
miR-203a: microRNA-203a
MMP: matrix metalloproteinase
NC: negative control
OA: osteoarthritis
PI: propidium iodide
qRT-PCR: quantitative real-time PCR
si-Smad3: small interfering RNA for Smad3
TGF-β: transforming growth factor-β
TNF-α: tumor necrosis factor α
